# Semiconducting to metallic transition with outstanding optoelectronic properties of CsSnCl_3_ perovskite under pressure

**DOI:** 10.1038/s41598-020-71223-3

**Published:** 2020-09-04

**Authors:** Jakiul Islam, A. K. M. Akther Hossain

**Affiliations:** Department of Physics, Bangladesh University of Engineering and Technology, Dhaka, 1000 Bangladesh

**Keywords:** Materials science, Mathematics and computing, Optics and photonics, Physics

## Abstract

Inorganic non-toxic metal halide perovskites have taken the dominant place in commercialization of the optoelectronic devices. The first principles simulation has been executed with the help of density functional theory to investigate the structural, optical, electronic and mechanical properties of non-toxic CsSnCl_3_ metal halide under various hydrostatic pressures up to 40 GPa. The analysis of optical functions displays that the absorption edge of CsSnCl_3_ perovskite is shifted remarkably toward the low energy region (red shift) with enhanced pressure. The absorptivity, conductivity and the value of dielectric constant also increases with the applied pressure. The investigation of mechanical properties reveals CsSnCl_3_ perovskite is mechanically stable as well as highly ductile and the ductility is increased with increasing pressure. The investigation of electronic properties shows semiconducting to metallic transition occurs in CsSnCl_3_ under elevated pressure. The Physics behind all these changes under hydrostatic pressure has been analyzed and explained in details within the available Scientific theory.

## Introduction

In recent years, metal halide perovskite materials of the renowned formula AMX_3_ (where, A = a cation, M = a metal ion, and X = a halogen anion) have attracted immense attention of the researchers due to their noticeable solar cell potency with extraordinary optoelectronic characteristics including wide range of absorption spectrum, enhanced optical absorption, tunable band gap, extended charge diffusion, high charge carrier mobility and low carrier effective masses^1,2^. The researchers established the application of these semiconducting materials are also wide in the field of electronic devices such as LEDs (Light Emitting Diodes), photodetector, and the devices which are extensively used for solar to fuel energy conversion^3–6^. Moreover, these metal halide perovskites are cheap and available in a large quantities on the earth. Consequently, these halide perovskite semiconductors would be more suitable and beneficial in solar cells application compared to the Si-based photovoltaic (PV) technology^1^. However, most of the perovskite halides with excellent properties comprise of lead (Pb) which is harmful for the environment^7–9^. Due to the environment contamination and world-wide energy crisis, clean and sustainable energy sources have taken great attention. Therefore, a large number of experimental and theoretical works have been performed by replacing Pb with a suitable metal cation in the last few years^10–13^. The study of mechanical properties reported by Roknuzzaman et al.^10^ demonstrates that the non-toxic CsSnCl_3_ perovskite has ductility entity but the halide perovskite semiconductor shows large band gap value (2.8 eV)^11^. As a result, the CsSnCl_3_ shows medium optical absorption and not appropriate for remarkable efficiency solar cells application. For this purpose, we have reported metal-doped CsSnCl_3_ to find a better Pb-free perovskite semiconductor for high potency solar cell application in previous work^14^. The major problem arises due to the generation of an intermediate band and consequently direct to indirect band gap transition in most of the best-entitled metal-doped halide perovskites^14,15^. Because, the indirect band gap may create phonons in the materials that may generate a heating effect to minimize the proficiency of the optoelectronic devices^16–22^. It was observed that pressure effect on halide perovskites has seized great attention by the researchers in recent years^23–28^, as it is generally known, effect of pressure has a vital role on the physical and chemical features of materials. The decrease of lattice volume of metal halides for the bulk phase is shown with enhanced pressure^25,28^. In a theoretical study of cesium tin halides, it is exhibited that band gap decreases with decreasing lattice parameter^29^. Applying hydrostatic pressure can reduce the lattice parameter. The goal of our present work is to apply various hydrostatic pressures on CsSnCl_3_ metal halide in order to reduce the band gap and consequently it may improve the optical absorption as well as proficiency of solar cells and other optoelectronic devices. Recently, external and internal pressure effects on cubic perovskites including CsSnCl_3_ have been reported in a theoretical work^30^. Shen et al. reported two first order phase transitions under high pressure up to 6 GPa, by using Raman scattering at room temperature. They discovered first phase transition (II/III) at 0.33 GPa, and another transition (III/IV) at 2.53 GPa^31^. A high temperature study by Voloshinovskii et al. reported that the CsSnCl_3_ crystal has a phase transition at 390 K from the monoclinic to the cubic^32^. This cubic and monoclinic phase transition is non-uniform throughout the crystal volume and both phases can coexist^32^. Ying et al. reported recently the tunable optical properties and topological non-trivial phase of CsSnCl_3_ along with other inorganic halide perovskites under pressure^33^. The present study deals with various hydrostatic pressure effects on the structural, electronic, optical and mechanical properties of CsSnCl_3_ metal halide using density functional theory (DFT) in details for the application in high proficiency solar cells and other probable optoelectronic devices.

## Computational methods

The ab-initio calculations have been carried out using DFT^34,35^ based plane wave pseudopotential technique as existed in CASTEP (Cambridge Serial Total Energy Package) module^36,37^. GGA (Generalized Gradient Approximation) was inserted in the simplified form of Perdew-Berke-Ernzerhof (PBE)^38^ for the evaluation of exchange correlation energy. The electron ion interaction was treated using ultrasoft pseudopotential of Vanderbilt model^39^. The optimized crystal structure was ensured employing BFGS (Broyden–Fletcher–Goldfarb–Shanno) technique^40^. The plane wave energy cutoff was settled at 550 eV with k-points 12 × 12 × 12 for obtaining the optimized structure and the properties calculations. The Brillouin zone sampling of k-points was executed using Monkhorst–Pack scheme^41^. The calculations of elastic constants were executed using finite strain theory^42^ as inserted in CASTEP. The strain amplitude was fixed at 0.003 as the optimum value. A scissor value (1.857 eV) was employed for the optical property calculations in order to compensate the difference between the value of theoretical band gap (0.943 eV) and experimental band gap (2.8 eV) of the CsSnCl_3_. The convergence thresholds were fixed as follows: total energy, 5 × 10^−6^ eV/atom; maximum displacements, 5 × 10^−4^ Å; maximum force, 0.01 eV/Å; maximum stress, 0.02 GPa.

## Results and discussion

### Structural properties

**Figure 1:**
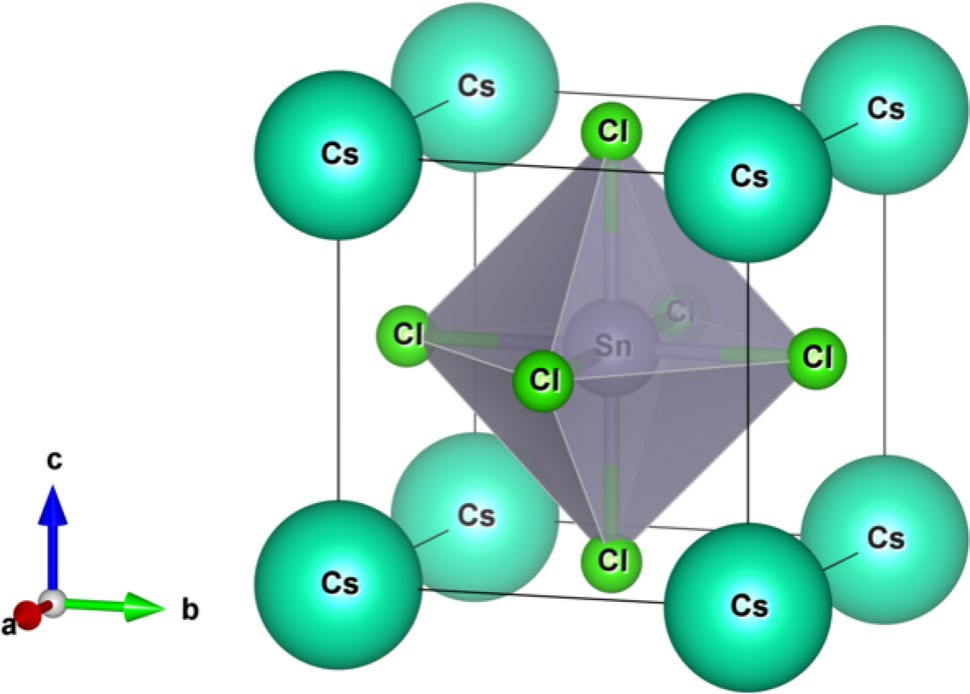
Constructed cubic crystal structure of CsSnCl_3_. The crystal structure was designed by using VESTA^43^.

**Figure 2:**
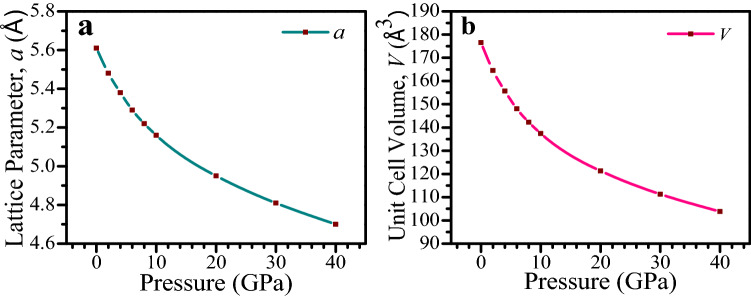
Variation of lattice constant (*a*) and unit cell volume (*V*) of cubic CsSnCl_3_ perovskite with pressure. The figures were drawn by using OriginPro 8.5 (https://www.OriginLab.com), taking DFT result from Material studio 7.

The CsSnCl_3_ semiconductor crystallizes in the cubic perovskite-type structure with space group *Pm* $\stackrel{-}{3}$*m* (no. 221). The cubic crystal structure of CsSnCl_3_ is drawn by using VESTA^43^, which is depicted in Fig. 1. The unit cell of the crystal consists of five atoms with one formula unit. The Cs atoms take place at corner with 1a Wyckoff position, the Sn atom possesses body centered site with 1b Wyckoff position, and the Cl atoms take place at face centered with 3c Wyckoff position^10^. The computed values of lattice parameter and the corresponding unit cell volume in this simulation with available experimental and theoretical results of the cubic CsSnCl_3_ are listed in Table 1. We have carried out the DFT study under various hydrostatic pressures from 0 to 40 GPa, with a step of 2 GPa up to 10 GPa, and then with a step of 10 GPa up to 40 GPa. The calculated lattice parameter at 0 GPa in this study exhibits very well consistent with previous theoretical works, bearing accuracy of this DFT work. The computed lattice parameter is slightly higher than the experimental findings and this is the general tendency of the GGA study. The influence of applied hydrostatic pressure on lattice parameter and cell volume is exhibited in Fig. 2a,b. From Fig. 2, it is observed that the values of lattice parameter and cell volume decrease in a smooth way with the increase of pressure, which implies that the space between atoms is getting reduced. As a result, repulsive influence between atoms become stronger, which conducts to the hardness of crystal compression under elevated pressure.

**Table 1:** The computed and the available experimental and theoretical values of lattice constant *a*, and the present evaluated unit cell volume *V* of CsSnCl_3_ at a different pressure.

Pressure (GPa)	*a* (Å)			*V* (Å^3^)
	This Work	Other Works	Experimental	
0	5.61	5.61^10^, 5.60^44^	5.56^45^, 5.57^12^	176.56
2	5.48	–	–	164.57
4	5.38	–	–	155.72
6	5.29	–	–	148.04
8	5.22	–	–	142.24
10	5.16	–	–	137.39
20	4.95	–	–	121.29
30	4.81	–	–	111.28
40	4.70	–	–	103.82

### Optical properties

The non-toxic CsSnCl_3_ metal halide shows less optical conductivity and medium optical absorption as reported in literature^10^. As a result, the CsSnCl_3_ perovskite is not suitable for a better efficiency solar cell application. Pressure can be a clean and effective thermodynamic approach to enhance the performance of CsSnCl_3_ as solar cell and other optoelectronic devices applications. The study of optical functions is very important fundamental approach to gain a deep knowledge about the compatibility of a material to better performance device applications. Therefore, in this current work we have investigated the crucial optical functions such as optical absorption, reflectivity, imaginary and real portion of dielectric functions, and optical conductivity of the cubic CsSnCl_3_ perovskite in details under various hydrostatic pressures up to 40 GPa.

**Figure 3:**
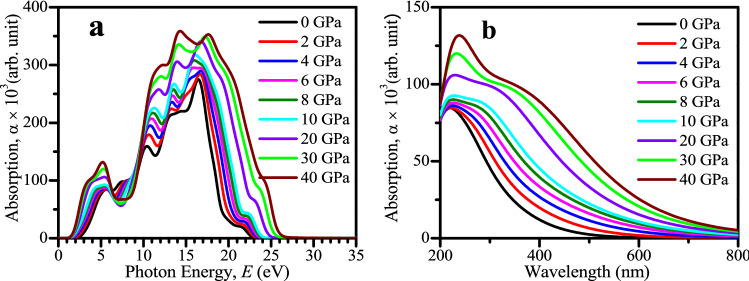
Simulated absorption profile of CsSnCl_3_ under pressure_._ (**a**) As a function of photon energy (eV) and (**b**) as a function of wavelength (nm). The absorption spectra was created by using Material studio 7 and drawn by using OriginPro 8.5.

The analyzed optical absorption profiles of CsSnCl_3_ perovskite are demonstrated in Fig. 3. The optical absorption coefficient is a crucial criteria to have knowledge about the capability of a material to absorb light energy and hence provides significant information about the solar energy conversion efficiency of the material which is required for the practical application of a material in prominent performance solar cell and other photovoltaic devices^14^. The optical absorption coefficient is stated as the measurement of penetration of light at specific energy (wavelength) into the material before being absorbed. Figure 3a demonstrates the absorption spectra of CsSnCl_3_ perovskite as a function of photon energy under different hydrostatic pressures up to 40 GPa. Figure 3a exhibits that the absorption edge of CsSnCl_3_ metal halide is shifted in the direction to the low energy region (red shift) with enhanced pressure. The redshift of absorption edge of CsSnCl_3_ under pressure, up to 8 GPa, is also observed by Ying et al. in literature^33^. The pressure-induced in CsSnCl_3_ enhances the absorption to a remarkable extent in the visible as well as in the ultraviolet region. The maximum broad absorption peak lies in the ultraviolet region which indicates that the studied CsSnCl_3_ metal halide would be an efficient material to make devices to sterilize surgical equipment. The maximum range of ultraviolet light energy absorption of a material indicates its potential application in surgical devices formation as sterilizing the devices made of such material becomes easier and efficient^46^.

The wavelength dependent absorption coefficient is exhibited in Fig. 3b for further clear understanding of the light absorbance nature of CsSnCl_3_ in the visible region under elevated hydrostatic pressure. According to the Fig. 3b, the CsSnCl_3_ shows very less absorption in the visible region under zero pressure but the value of absorption coefficient becomes high to a greater extent with applied pressure. As a result, the non-toxic CsSnCl_3_ metal halide under pressure would be a better replacement of toxic Pb-containing materials for the application in remarkable potency solar cells and other optoelectronic devices. The reason behind the increase of absorption coefficient with applied pressure has been discussed in electronic properties section (“Electronic properties”) in details.

**Figure 4:**
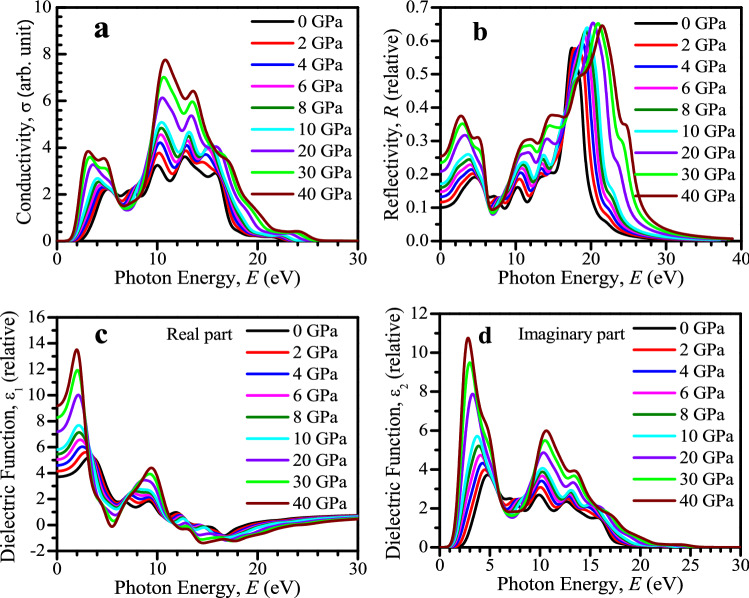
Calculated pressure-induced spectra of (**a**) optical conductivity, (**b**) reflectivity, (**c**) real portion of dielectric function, and (**d**) imaginary portion of dielectric function of CsSnCl_3_. All the optical functions were obtained from Material studio 7 and drawn by using OriginPro 8.5.

The optical conductivity is basically another form of photoconductivity^47^. The amount of photoconductivity as well as electrical conductivity enhances because of increasing photons absorption. The conductivity spectra (real portion) under several hydrostatic pressures is illustrated in Fig. 4a up to 30 eV of photon energy. The conductivity spectra has similar characteristics like absorption spectra as shown in Fig. 3a, because, material releases free carriers for conduction when it absorbs energy. The optical conductivity enhances with applied pressure which is a result of the enhancing absorption coefficient (see Fig. 3) with increased pressure.

The surface nature of the CsSnCl_3_ perovskite can be understood in terms of reflected light energy from the surface^14^. Figure 4b demonstrates the reflectivity spectra of the CsSnCl_3_ metal halide for photon energy up to 40 eV under various applied pressures. The amount of reflectivity increases as much as enhanced pressure which may a cause to reduce the potency of the solar cell. Therefore, further study should be operated to minimize the reflectivity of the pressure-induced CsSnCl_3_ perovskite in the visible energy area which may improve further the absorptivity as well as solar cell efficiency.

The act of a material in reaction to incident light energy is termed as dielectric function. The static peak of dielectric function is an efficient parameter which provides useful knowledge about the charge carrier recombination rate and hence the entire potency of optoelectronic devices^48^. The materials which have improved value of dielectric constant means the materials have low charge carrier recombination rate and greater efficiency of the optoelectronic devices. Figure 4c,d display the real and imaginary part of dielectric constant, respectively, of the pressure-induced CsSnCl_3_ for light energy up to 30 eV. The static peak of dielectric constant of both real and imaginary parts of the CsSnCl_3_ perovskite rises in the visible region with enhanced pressure as depicted in Fig. 4c,d. The materials of large band gap generally exhibit low static value of dielectric constant^49^. Therefore, pressured-induced CsSnCl_3_ metal halide exhibits higher static value of dielectric constant as band gap is decreased (see electronic properties segment) with pressure. The imaginary part of the dielectric function is associated directly with the material band structure and explains its absorption nature^13^. The peaks of the imaginary part of dielectric functions are increased in a prominent way with pressure and shift to the low energy region, which justifies the result of absorption spectra as illustrated in Fig. 3a. However, at high energy region (above 26 eV) for all the pressured-induced CsSnCl_3_ samples, the imaginary segment of dielectric constant goes to zero and the real part reaches to unity approximately. This result means that all the pressure-induced samples reveal high transparency and consequently little absorption in the high energy region (above 26 eV), which is also evident from the profile of absorption coefficient as given in Fig. 3a.

### Electronic properties

**Figure 5:**
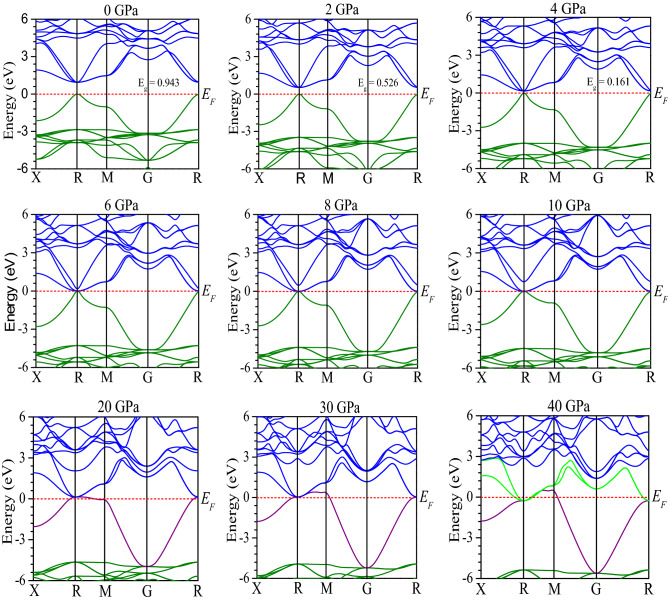
Computed band structure of CsSnCl_3_ under pressure using GGA method. All the band structures were calculated by using Material studio 7 and drawn by using OriginPro 8.5.

The analysis of key electronic properties (band structure and density of states) is very important to gain deep understanding of the optical functions. The studied band structures of CsSnCl_3_ under variant pressures are demonstrated in Fig. 5. The Fermi level (*E*_*F*_) is exhibited at zero of photon energy scale which is presented from − 6 eV to + 6 eV for all the samples. According to semi-conductive theory, the material band structure close to the *E*_*F*_ is very significant criteria to gain knowledge about the physical nature of the material. Therefore, we have exhibited the band structure configuration around the Fermi level. Figure 5 demonstrates that the band gap of CsSnCl_3_ without any external pressure at R-point of Brillouin zone is 0.943 eV (direct band gap), the same value of band gap is observed by Roknuzzaman et al.^10^, carrying novelty of this current GGA work. It is explicit that the evaluated band gap of GGA approach underestimates the band gap value (2.8 eV) of experimental work^11^. This is well-known general limitation of the GGA approach. The limitation of band gap underestimation is also found in LDA + U and LDA techniques. To overcome this error in calculating band gap, researchers have introduced some techniques such as GW method^50^, hybrid functional^51^, but these techniques also comprise of some limitations. GGA + U technique^52^ is used in some purposes to execute partial correction of the theoretically assessed band gap in comparison with experimentally attained value of band gap. However, recently Nayak and his coworkers reported that the overall behavior of the variation in the band gap (*E*_*g*_) and the band structure with pressure were independent of functional employed and that the PBE approach provided reasonably exact results, which suggested the use of PBE/GGA functional for pressure study on materials^53,54^. Therefore, we have investigated the band structure of the CsSnCl_3_ perovskite using GGA along with PBE. The band structure has been computed with a step of 2 GPa up to 10 GPa, and then with a step of 10 GPa up to 40 GPa. Figure 5 exhibits that as the pressure is going up, the valance band maxima and conduction band minima at R-point start to shift toward *E*_*F*_. As a result, the *E*_*g*_ of CsSnCl_3_ decreases with pressure and consequently becomes metallic (overlap of valance band and conduction band at *E*_*F*_) at a certain pressure. Overlap of valance and conduction band of a semiconducting material under a certain pressure indicates the semiconductor to metal transition at that pressure^53^. As band gap of the CsSnCl_3_ is reduced and consequently vanished with increase of pressure, then the transition of excited electron becomes much more convenient and faster from valance band to conduction band. As a result, the CsSnCl_3_ metal halide has increasing affinity of absorption coefficient in the visible region with increasing applied hydrostatic pressures as depicted in Fig. 3. However, though no band gap of the CsSnCl_3_ at 6 GPa, 8 GPa, and 10 GPa is observed in naked eyes, but the band gap is in critical stage which is also revealed by further GGA-PBE calculation with scissor value (see Supplementary Fig. 1). For CsSnCl_3_ metal halide the band gap underestimation is found in our calculation is about 1.857 eV as compared between the theoretical band gap (0.943 eV) and the experimental band gap (2.8 eV). For further clear understanding, we have calculated the band structure taking into account the underestimated value of the band gap in PBE calculation. We have used a scissor value (1.857) in purpose to compensate the band gap underestimation between theoretical (0.943 eV) and experimental observation and the observed band structures have been attached as supplementary file (see Supplementary Fig. 1). Figure 5 demonstrates that the valance band and conduction band significantly overlap at the Fermi level under pressure at 20 GPa, which indicates the metallic nature of CsSnCl_3_.

**Figure 6:**
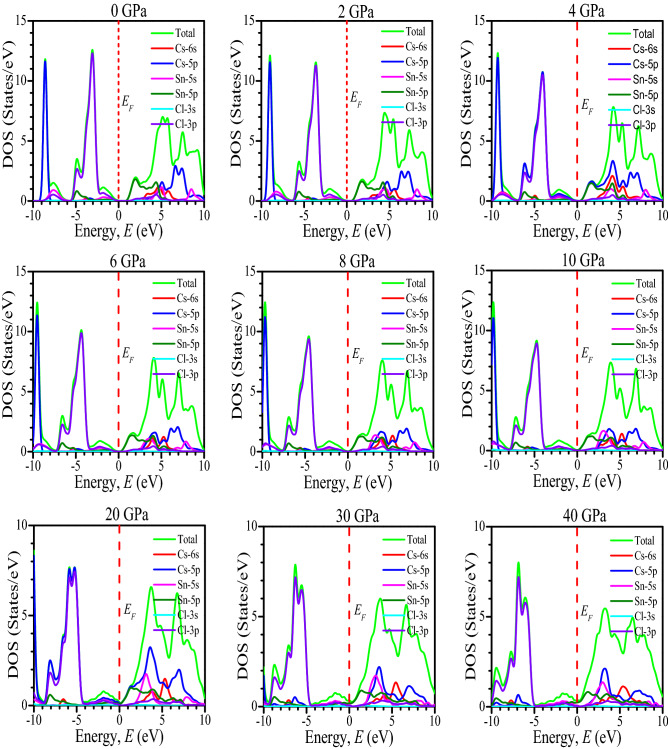
Calculated TDOS and PDOS of non-toxic CsSnCl_3_ under pressure. The DOS profiles were drawn by using OriginPro 8.5, taking DFT result from Material studio 7.

**Figure 7:**
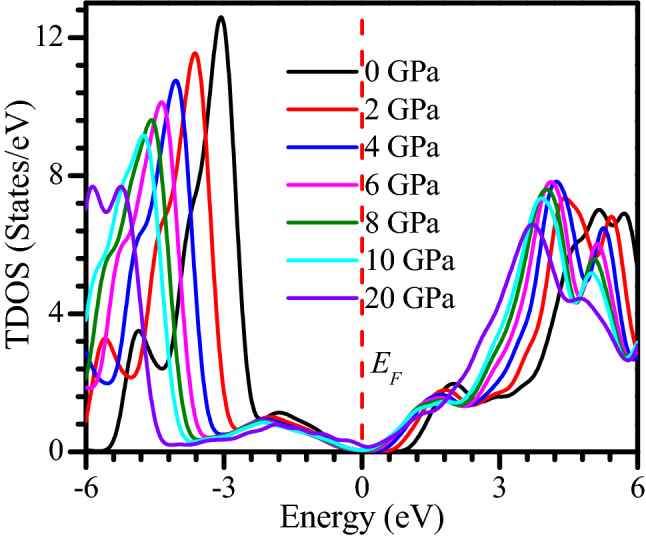
DOS value of CsSnCl_3_ near to the Fermi level. The figure was drawn by using OriginPro 8.5, taking DFT result from Material studio 7.

The metallic nature of the CsSnCl_3_ under pressure has been further well-understood by the investigation of density of states (DOS). The total density of states (TDOS) and partial density of states (PDOS) of the CsSnCl_3_ perovskite under several applied pressure is plotted in Fig. 6. Figure 6 depicts that the TDOS below *E*_*F*_ (valance band) is mainly originated by Cl-3p orbital with some contribution of Cs-5p, Sn-5s, and Sn-5p orbitals for the all studied samples of the CsSnCl_3_ under hydrostatic pressure or at zero hydrostatic pressure, without two exceptions at 4 GPa and 20 GPa. At 4 GPa and 20 GPa, the valance band near *E*_F_ is approximately equally contributed by Cl-3p and Cs-5p orbitals. The TDOS above *E*_*F*_ (conduction band) is mostly originated by Sn-5p orbitals with small contribution of Cs-6s, Cs-5p, and Sn-5s orbitals for all phases of the CsSnCl_3_ metal halide, without two exceptions at 4 GPa and 20 GPa as plotted in Fig. 6. At 4 GPa and 20 GPa, the Cs-5p orbital contribution becomes higher near to the *E*_*F*_ of the conduction band. The non-zero value of DOS at the Fermi level indicates the metallic nature of a material^55,56^. Figure 6 demonstrates that at 0 GPa, 2 GPa, and 4 GPa, the value of TDOS is zero at Fermi level which indicates the semiconducting nature of the CsSnCl_3_. Whereas, at pressure 6 GPa, 8 GPa and 10 GPa, a negligible value of TDOS is appeared at Fermi level as exhibited in Fig. 6. However, the value of TDOS is non-zero and significant at 20 GPa, which reveals the semiconducting-metallic transition of the CsSnCl_3_ metal halide at this pressure. For further clear understanding, we have exhibited the value of TDOS near to the Fermi level in Fig. 7. It can been noticed that the DOS at the Fermi level is significantly appeared at 20 GPa and has increasing affinity with increasing pressure, indicating the semiconducting-metallic transition of the CsSnCl_3_ metal halide under elevated pressure. . The present study predicts the semiconducting to metallic transition in CsSnCl_3_ under elevated pressure with a detailed investigation of band structure as well as density of states. However, though, Nayak and his coworkers found the overall variation of the behavior of the band gap and band structure with pressure approximately independent of the functional used for their studied materials but the transition pressure of this PBE calculation for semiconductor to metal transition in CsSnCl_3_ may vary slightly with experimental observation. Because this overall variation of the band gap and band structure with pressure may vary depending on the materials. Therefore, future experimental investigation should be carried out to locate the exact transition pressure for semiconductor to metal transition in CsSnCl_3_. We believe this study would be efficient enough for future experimental investigation to locate the exact transition pressure for semiconducting to metallic transition of CsSnCl_3_ metal halide.

### Mechanical properties

The elastic constants of solid materials are crucial parameters as they provide significant link between the mechanical properties and fruitful information concerning the characteristic of existing forces in solids and particularly for the material stability and stiffness^10,57^. Elastic constants also provide dynamic information about the ability of a crystal to resist external pressure. As lattice parameter reduces with pressure (see Fig. 2), it is very significant to investigate the influences of pressure on the elastic constants for the purpose of understanding the mechanical properties of the CsSnCl_3_ metal halide. The cubic CsSnCl_3_ metal halide comprises of three distinguished elastic constants, *C*_11_, *C*_12,_ and *C*_44_, the obtained results of the elastic constants in the present DFT work under variant pressures with available other theoretical result are listed in Table 2. The mechanical stability of a cubic crystal is determined by renowned Born stability criteria which is expressed as:

$$ C_{11} > 0,\;C_{44} > 0,\;C_{11} + 2C_{12} > 0\;{\text{and}}\;C_{11} - C_{12} > 0 $$

**Table 2:** The calculated values of *C*_ij_ (GPa) and Cauchy pressure *C*_12_–*C*_44_ (GPa) of cubic CsSnCl_3_ perovskite under variant pressure.

Pressure (GPa)	*C*_11_	*C*_12_	*C*_44_	*C*_12_–*C*_44_
0 (Ref. 10)	50.66	8.71	6.01	2.70
0	50.63	8.80	6.15	2.65
2	69.99	12.34	6.09	6.25
4	86.37	14.52	5.51	9.01
6	104.12	18.37	5.79	12.58
8	121.67	21.42	5.95	15.47
10	140.45	22.62	5.46	17.16
20	206.03	42.54	5.04	37.50
30	281.98	58.39	2.83	55.56
40	348.21	72.88	3.30	69.58

Table 2 shows that the CsSnCl_3_ metal halide is mechanically stable under considerable variant pressure as satisfies the above stability criteria. Moreover, the present computed values of elastic constants and other mechanical properties (see Table 3) at zero pressure are very well matched with previous available DFT result^10^, bearing nicety of the present calculation. From Table 2, it can be observed that the values of *C*_11_ and *C*_12_ increase rapidly with pressure going up, whereas the value of *C*_44_ remains invariant almost under pressure, up to 40 GPa. The elastic constants *C*_11_ and *C*_12_ are connected with the elasticity in length, increase with pressure enhanced^58^. Whereas, *C*_44_ is connected with the elasticity in shape, which provides relation between the deformation in shape and the stiffness^58^. The Cauchy pressure (*C*_12_−*C*_44_) is a well-known parameter to indicate the ductile and brittle characteristics of materials. The negative (positive) value of Cauchy pressure of a material indicates its brittle (ductile) nature. From Table 2, it can be seen that the Cauchy pressure value of the CsSnCl_3_ perovskite under all studied pressure is positive as zero pressure and increases with pressure enhanced, which indicates the ductile nature of the perovskite increases with pressure enhanced. The mechanical properties such as Bulk modulus, *B*, Shear modulus, *G*, Young’s modulus, *E*, Pugh’s ratio, *B/G*, and Poisson’s ratio, *v,* of the cubic CsSnCl_3_ metal halide are calculated with the help of well-known expressions as given in literature^14^ and listed in Table 3. The lower values of *B*, *G* and *E* of the CsSnCl_3_ under zero pressure indicates it’s as soft material. From Table 3, it can be noticed that the values of *B*, *G*, and *E* rises with increased pressure, which indicates the applying hydrostatic pressure provides benefit to the hardness of CsSnCl_3_. Ying et al.^33^ also showed that the values of elastic moduli of CsSnCl_3_ increase with enhanced pressure and they had shown this phenomena up to 8 GPa, indicating reliability of the present DFT results. The Pugh’s ratio is an important factor to indicate ductile and brittle behavior of a crystal. The low (high) value of *B/G* indicates the brittle (ductile) nature of the material and the critical value is considered as 1.75^59^.

**Table 3:** The calculated mechanical properties of CsSnCl_3_ at a different pressure.

Pressure (GPa)	*B* (GPa)	*G* (GPa)	*E* (GPa)	(*B/G*)	*v*
0 (Ref.^10^)	22.70	10.20	26.61	2.22	0.30
0	22.74	10.31	26.88	2.21	0.303
2	31.56	12.04	32.04	2.62	0.331
4	38.47	13.00	35.05	2.96	0.348
6	46.95	14.74	40.03	3.19	0.358
8	54.84	16.41	44.76	3.34	0.364
10	61.90	17.71	48.49	3.50	0.369
20	97.04	21.89	61.08	4.43	0.395
30	132.92	25.53	71.98	5.21	0.410
40	164.66	31.23	88.12	5.27	0.411

**Figure 8:**
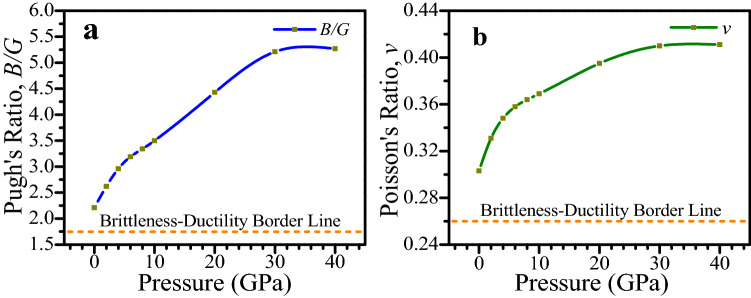
(**a**) Variation of Pugh’s ratio and (**b**) variation of Poisson’s ratio of CsSnCl_3_ perovskite at a different pressure. The figures of mechanical properties were drawn by using OriginPro 8.5, taking DFT result from Material studio 7.

Table 3 shows that the *B/G* value of CsSnCl_3_ metal halide under zero pressure is greater than the critical value which reveals the ductile nature of the metal halide. From Table 3, it is also noticed that the *B/G* value increases with the increase of pressure, which indicates that the ductility of CsSnCl_3_ can be improved by the rise of pressure. The *v* is very useful criteria which provides fruitful knowledge about the bonding forces and stability of a crystal. The maximum value and minimum value of *v* for existing central forces in ionic crystals are considered as 0.5 and 0.25, respectively^60^. Ionic crystal’s interatomic forces are central forces^60^. From Table 3, it can be noticed that the value of *v* of CsSnCl_3_ metal halide at ambient condition is 0.303 which is lower than 0.5 but greater than 0.25, indicating the existence of central forces in the metal halide. The value of *v* increases with increasing pressure. After 20 GPa, the value of *v* is not increased significantly (see Table 3), which indicates the strong central forces exist in CsSnCl_3_. The Poisson’s ratio is also a useful indicator of brittleness and ductility of materials. The critical value of *v* to indicate ductile and brittle behavior of a material is 0.26^14^. The value of *v* of CsSnCl_3_ perovskite without any external pressure is higher than 0.26 as displayed in Table 3, which reveals the ductile characteristics of the perovskite. The value of *v* increases with the increase of pressure, which predicts that the ductility can be improved further by applying external pressure. The variation of *B/G* and *v* of CsSnCl_3_ perovskite with pressure have been displayed in Fig. 8a,b, respectively, for further clear understanding the ductile behavior of the perovskite. From Fig. 8, it is evident that the ductility of CsSnCl_3_ increases with enhanced pressure and hence pressure can be an efficient approach where high ductility is required to fabricate devices of CsSnCl_3_.

## Conclusions

In brief, structural, elastic, optical, and electronic properties of cubic CsSnCl_3_ metal halide under hydrostatic pressure have been studied using DFT-based CASTEP module. The lattice constant and cell volume of the CsSnCl_3_ decrease with pressure. The elastic moduli increase with pressure, which benefits to the hardness of CsSnCl_3_. The study of Poisson’s ratio and Pugh’s ratio shows that the CsSnCl_3_ material has increasing affinity of ductility with increasing pressure and the material can be efficient for practical devices application where high ductility is needed. The band gap decreases with pressure and consequently semiconducting to metallic transition occurs in CsSnCl_3_ under elevated pressure. The optical absorption as well as conductivity increase remarkably in the visible region with enhanced pressure, which indicates that the performance of CsSnCl_3_ perovskite solar cell and other optoelectronic devices can be improved greatly by inducing pressure. The tricks provided in this study would be effective to property investigations of other types of perovskites and also for other kinds of materials for modeling devices with outstanding photovoltaic and optoelectronic performance.

## Supplementary information

Supplementary file1

Supplementary file2
