# Supplementary material for: Semiconducting to metallic transition with outstanding optoelectronic properties of CsSnCl3 perovskite under pressure
Source: Sci Rep. 2020 Sep 4;10:14391. doi: 10.1038/s41598-020-71223-3 (PMC7474070; doi:10.1038/s41598-020-71223-3)
Supplement: Supplementary file 1 — Supplementary file1 [file 41598_2020_71223_MOESM1_ESM.docx]

**Semiconducting to metallic transition with outstanding optoelectronic properties of CsSnCl_3_ perovskite under pressure**

Jakiul Islam* and A.K.M. Akther Hossain

Department of Physics, Bangladesh University of Engineering and Technology, Dhaka-1000, Bangladesh.

***Electronic band structure analysis***

From the supplementary Figure 1, it is easily observable that the band gap is decreased with enhanced pressure but at and above 6 GPa and below 20 GPa we found the band gap remains the same which we named as critical stage of band gap. At 20 GPa, the band gap is vanished and the overlap of the valance band and conduction band occurs. As a result we found the significant value of density of states at this pressure (see Figure 6 and Figure 7). The overlap of valance and conduction band and the significant value density of states at 20 GPa indicate the semiconductor-to metal transition in CsSnCl_3_ at this pressure. Future experimental study should be conducted to find the exact transition pressure for semiconductor to metallic transition in CsSnCl_3_. The present detailed analysis on band structures and DOS would be helpful enough for future experimental and theoretical pressure study on CsSnCl_3_.

**Supplementary Figure 1**.

**Supplementary Figure caption:**

**Figure 1.** Calculated electronic band structure of CsSnCl_3_ metal halide under several hydrostatic pressures using GGA-PBE functional with a scissor value (1.857).
