# Supplementary material for: Semiconducting to metallic transition with outstanding optoelectronic properties of CsSnCl3 perovskite under pressure
Source: Sci Rep. 2020 Sep 4;10:14391. doi: 10.1038/s41598-020-71223-3 (PMC7474070; doi:10.1038/s41598-020-71223-3)
Supplement: Supplementary file 2 — Supplementary file2 [file 41598_2020_71223_MOESM2_ESM.docx]

**Semiconducting to metallic transition with outstanding optoelectronic properties of CsSnCl_3_ perovskite under pressure**

Jakiul Islam and A.K.M. Akther Hossain

Department of Physics, Bangladesh University of Engineering and Technology, Dhaka-1000, Bangladesh.

***Optical properties analysis***

As no band gap is observed under high pressure (see Fig. 5, 7 and supplementary Fig.1) that is why we have further investigated the fundamental optical functions such as optical absorption and conductivity not using any scissor value. It is explicit from supplementary Fig. 2 that both absorptivity and conductivity edges shift to the low energy region (red shift) with increasing pressure and start at zero photon energy under high pressure which indicates that the semiconducting CsSnCl_3_ metal halide have metallic nature under high pressure.

**Supplementary Figure 2**

**Supplementary Fig. 2 caption:**

**Supplementary Fig. 2.** Calculated optical functions of CsSnCl_3_ using GGA-PBE functional without scissor value, (a) absorptivity and (b) conductivity. The figure was drawn using Origin pro 8.5, taking DFT result from Material studio 7.
